# The rs10455872-*G* allele of the *LPA* gene is associated with high lipoprotein(a) levels and increased aortic valve calcium in a Mexican adult population

**DOI:** 10.1590/1678-4685-GMB-2017-0371

**Published:** 2019-11-14

**Authors:** Guillermo Cardoso-Saldaña, José Manuel Fragoso, Shamar Lale-Farjat, Margarita Torres-Tamayo, Carlos Posadas-Romero, Gilberto Vargas-Alarcón, Rosalinda Posadas-Sánchez

**Affiliations:** 1 Department of Endocrinology, Instituto Nacional de Cardiología - Ignacio Chávez, México City, México.; 2 Department of Molecular Biology, Instituto Nacional de Cardiología - Ignacio Chávez, México City, México.

**Keywords:** Aortic valve calcification, genetic susceptibility, *LPA* gene polymorphism

## Abstract

Polymorphisms in the *LPA* gene have been associated with aortic valve calcification (AVC). There are wide differences in the allelic frequencies, Lp(a) levels, and the association with AVC among ethnic groups. The aim of this study was to determine the association of the *LPA* gene polymorphisms with Lp(a) levels and risk of developing AVC, in Mexican-Mestizos population. Six *LPA* polymorphisms (rs10455872, rs7765803, rs6907156, rs1321195, rs12212807 and rs6919346) were genotyped by TaqMan assays in 1,265 individuals without premature coronary artery disease. The presence of AVC was determined by computed tomography. The association of the *LPA* polymorphisms with AVC, Lp(a), and other cardiovascular risk factors (CVRF) was evaluated using logistic regression analysis. Compared to AA genotype, subjects with AG+GG genotypes had high prevalence of Lp(a) ≥ 30 mg/dL (7.1% vs. 23.7%, *p*<0.001) and AVC (19.0% vs. 29.4%, *p*=0.007). In a model adjusted for several CVRF, the *LPA* rs10455872-G allele was associated with high Lp(a) levels and AVC. Carriers of G allele had a high risk of Lp(a) ≥ 30 mg/dL (OR= 3.86, CI 95%: 2.2 - 6.7, *p*=0.001) and AVC (OR= 2.54, CI 95%: 1.56 - 4.14, *p*=0.001), independently of other CVRF. In this population, carriers of rs10455872-G allele had 3.86 and 2.54 higher risk of Lp(a) ≥ 30 mg/dL or presence of AVC, respectively.

## Introduction

Lipoprotein(a) [Lp(a)] is a low density lipoprotein (LDL) bound to apo(a), a polymorphic glycoprotein of high molecular weight (300 to 800 kDa) very similar to plasminogen (McLean *et al.*, 1987; Guevara *et al.*, 1992). A high concentration of Lp(a) is a documented risk factor for coronary artery disease (CAD) (Danesh *et al.*, 2000; Nordestgaard *et al.*, 2010).

Aortic valve calcification (AVC) constitutes one of the first stages of valvular disease that can obstruct blood flow (Kamstrup *et al.*, 2014). This condition has been found in 2% to 7% of the population older than 65 years of age (Aronow *et al.*, 1999), and can reach 40% in individuals with other atherosclerosis risk factors, like smoking, diabetes, hypertension, and dyslipidemia (Taylor *et al.*, 2005). Ethnicity also influences importantly the prevalence of this disease (Nasir *et al.*, 2010; Smith *et al.*, 2014). AVC is an early marker of valvular disease and coronary atherosclerosis as it is associated with a 50% increase in the risk of myocardial infarction and cardiovascular mortality (Otto *et al.*, 1999).

In the Mexican population without evidence of CAD, prevalence of AVC is of 20%, and is significantly associated with traditional coronary risk factors (Acuña-Valerio *et al.*, 2017)

Several studies have described an independent and significant association of high Lp(a) concentrations with the presence of AVC (Rajamannan *et al.*, 2011; Kamstrup *et al.*, 2014; Yutzey *et al.*, 2014). However, this association changes widely among ethnic groups (Thanassoulis *et al.*, 2013) probably due to different polymorphisms of some genes, like the *LPA*, the direct influence of Lp(a) concentration, and the risk of aortic valve stenosis (Bossé *et al.*, 2008). These observations point out the relevance of considering ethnicity when studying the association between Lp(a) and AVC. Previous reports have described Lp(a) concentrations (Cardoso-Saldaña *et al.*, 1997), apo(a) isoforms (Cardoso-Saldaña *et al.*, 2006) and their association with atherosclerosis in Mexican subjects with and without CAD (Baños-González *et al.*, 2010). However, in Mexican-Mestizos, the possible relation of the *LPA* gene variants to the concentration of Lp(a) in plasma and the presence of AVC is not known. Hence, the aim of the present study was to evaluate the role of *LPA* gene polymorphisms, as susceptibility markers for AVC in a population of adult Mexican-Mestizos and whether there is an association with increased concentrations of Lp(a).

## Materials and Methods

### Subjects

We included 1,265 subjects of the Genetics of the Atherosclerotic Disease (GEA, for its initials in Spanish) Mexican Study, designed at the *Instituto Nacional de Cardiología “Ignacio Chavez”* in Mexico City to investigate the association of genetic factors with traditional and emerging cardiovascular risk factors in the Mexican adult population (Villarreal -Molina *et al.*, 2012). All GEA participants were Mexican-Mestizos, that is, at least the last three generations have been born in Mexico, and only one person per family was included in the study. Participants were selected from volunteers donating blood at the blood bank, or recruited through invitation among the population attending health community centers in the metropolitan area of Mexico City. We selected individuals from 30 to 75 years of age, without a family and personal history of premature CAD, without acute or chronic inflammatory processes, or a history or clinical evidence of renal (serum creatinine > 1.5 mg/dL) or liver (viral hepatitis or drug-induced) diseases. Individuals with cancer or under corticosteroids treatment were not included. The study protocol was approved by the institutional Ethics Committee and performed according to the Helsinki Declaration guidelines. All participants signed an informed consent letter.

Validated questionnaires were applied to the participants to obtain demographic, family and personal history of cardiovascular risk, physical activity, and use of pharmaceuticals information. Weight was determined in kilograms (kg) and height in centimeters (cm) using a calibrated scale and a wall stadiometer. Body mass index (BMI) was calculated with the formula: weight (kg)/height (m^2^). Waist was measured with a fiberglass metric measure, at the midpoint of the distance between the lower part of the last rib and the iliac crest with a 0.5-cm approximation. Systolic and diastolic arterial pressure was measured three times, in sitting position after at least 5 min rest, and the average of the last two consecutive measurements was considered for the analysis.

### Biochemical analysis

Blood samples were obtained from an antecubital vein after a 10-h fasting period and 20 min in sitting position. Glucose, total cholesterol, triglycerides, and high density lipoprotein cholesterol (HDL-C) concentrations were determined in the plasma with enzymatic methods (Roche/Hitachi, Germany) in a Hitachi 902 auto-analyzer (Hitachi LTD, Tokyo Japan). Low density lipoprotein cholesterol (LDL-C) was calculated with the Friedewalds formula modified by DeLong *et al.* (1986), the non-HDL cholesterol was calculated subtracting the HDL-C from the total cholesterol. Reproducibility and accuracy of lipids and lipoproteins determinations were periodically evaluated by the Lipids Standardization Program of the Center for Disease Control and Prevention of the USA (LSP-CDC, Atlanta, GA. USA). Intra- and inter-assay variation coefficients were below 3%. Lp(a) concentrations was determined through immunonephelometry with the N Latex Lp(a) reagent (SIEMENS, Health Care Diagnostics Products, GmbH, Marburg, Germany) (Marcovina *et al.*, 2000), in an automatized BN ProSpec® equipment following the manufacturer’s instructions. Intra- and inter-assay variation coefficients were below 6%.

### Computed tomography study

Coronary artery calcification and aortic valve calcification expressed in Hounsfield units (HU) were assessed through 64-detector helical tomography (Somaton Sensation, Siemens, Malvern, PA, USA) with cardiac synchronization by means of a prospective protocol with the following parameters: 120 kV, 120 mA, and 3-mm slices according to the Agatston method (Mautner *et al.*, 1994). This method correlates significantly with the calcium mass measured directly in the valves (Budoff *et al.*, 2002; Messika-Zeitoun *et al.*, 2004). The aortic wall calcium, found directly connected to the valve’s calcium, was included in the AVC score. Images were interpreted by experienced radiologists. The intra-observed variability for AVC was analyzed in 20 random cases, and the correlation coefficient was of 0.99 with *p* < 0.001.

### Genetic Analysis

DNA extraction was performed from blood peripheral as described by Lahiri and Nurnberger (1991). The rs10455872, rs7765803, rs6907156, rs1321195, rs12212507 and rs6919346 SNPs of the *LPA* gene were genotyped using 5exonuclease TaqMan assays on an ABI Prism 7900HT Fast Real-Time PCR System, according to the manufacturers recommendations (Applied Biosystems, Foster City, USA). For each polymorphism, we identified the CAD-risk allele or the minor allele frequency (MAF); subjects carrying the risk allele were compared to homozygotes carrying the wild-type allele.

Because the Mexican-Mestizo population is admixed, in order to assess the possible influence of population stratification, a panel of 265 ancestry informative markers (AIMs) distinguishing mainly Amerindian, European and African ancestry were selected (Silva-Zolezzi *et al.*, 2009) and genotyped on Illumina BeadStation using the GoldenGate assay. Duplicate control samples were genotyped on each chip, which also served as internal controls for quality of clustering and reproducibility. The primary analysis of the genotyping data with the Illumina Genome Studio software v.2011.1 was followed by visual inspection and assessment of data quality and clustering. Genotyping accuracy was also assessed by genotype clustering using the Illumina GeneTrain score, which is a measure of the clustering confidence of individual SNP alleles. Global Caucasian, Amerindian and African ancestry were determined in each individual using the ADMIXTURE software.

### Statistical analysis

Data are expressed as mean and standard deviation, or median and interquartile range for continuous variables, and as frequencies and percentages for categorical variables. Comparisons for continuous variables were performed with Student’s *t* or Mann-Whitney U tests*,* according to variable distribution, and by chi-square tests for categorical variables. CAC and AVC were analyzed as categorical variables (CAC > 0; AVC > 0 HU). Allelic and genotypic frequencies of the polymorphisms of the analyzed molecules were obtained through direct counting, and the Hardy-Weinberg equilibrium was assessed by chi-square analysis. Multivariate logistic regression analysis was used to investigate the independent association between each *LPA* polymorphisms with the presence of Lp(a) ≥ 30 mg/dL, the presence of CAC, or AVC > 0 HU. Multiple logistic regression models were constructed including one variable at the time, and the final model included variables with biological relevance or with statistical significance. A *p-*value <0.05 was considered significant. Analyses were made with the statistics software SPSS V 16.0.

## Results

Of the 1,265 studied subjects, with a mean age of 53.3 ± 9.4 years, 53% were women and 47% men. Prevalence of Lp(a) of ≥ 30 mg/dL, CAC and AVC > 0 HU were 9.2%, 26.6%, and 20.0%, respectively. In addition, 11.1% of the study population presented hypertension, 13.7% had diabetes mellitus, and 21.9% were active smokers (Table 1). Global ancestry was 54.0%, 35.8%, and 10% of Native American, Caucasian and African ancestry, respectively. The polymorphisms were in Hardy-Weinberg equilibrium (HWE, *p*>0.05). The MAF for rs10455872 *A*>*G*, rs7765803 *C*>*G*, rs6907156 *T*>*C*, rs1321195 *G*>*A*, rs12212507 *A*>*G* and rs6919346 *C*>*T* polymorphisms were 0.087, 0.067, 0.012, 0.128, 0.06 and 0.008, respectively. Three of the six studied polymorphisms, rs10455872-*G* (*p*=0.013), rs6907156-*T* (*p*=0.021), and rs7765803-*G* (*p*=0.001) were associated with high Lp(a) concentrations. Only the rs10455872-*G* polymorphism was associated with AVC > 0 HU (*p*=0.013). No other significant association was observed between the studied *LPA* polymorphisms with clinical or biochemical variables (data not shown). The population was divided into two groups according to the rs10455872 *LPA* gene variant: one included the wild-type genotype (*AA*) carriers and the other the carriers of the *GA* and *GG* genotypes.

**Table 1:** Characteristics of the Mexican-Mestizo population according to *LPA* rs10455872 genotype.

	Genotypes for *LPA* rs10455872			
	Total n =1265	*AA* n =1146	*AG + GG* n =119	*p*
Age (years)	53.3 ± 9.4	53.8 ± 9.1	51.2 ± 10.6	**0.011**
Waist (cm)	94.811.5.0	94.9 ± 11.5	94.4 ± 11.5	ns
BMI (kg/m^2^)	28.5 ± 4.4	28.5 ± 4.4	28.6 ± 4.7	ns
Systolic blood pressure (mmHg)	117.3 ± 17.5	117.9 ± 18.0	116.8 ± 17.7	ns
Diastolic blood pressure (mmHg)	72.2 ± 9.4	72.4 ± 9.6	72.0 ± 10.0	ns
Total cholesterol (mg/dL)	193.1 ± 37.3	193.1 ± 37.3	193.3 ± 33.4	ns
HDL-C (mg/dL)	46.0 ± 13.4	46.3 ± 13.4	47.3 ± 14.7	ns
Triglycerides (mg/dL)	147.5(112-201.0)	148.0(112.9-201.0)	138.6(97.0-202.0)	ns*
LDL-C (mg/dL)	118.1 ± 31.8	118.1 ± 31.9	118.9 ± 30.4	ns
Glucose (mg/dL)	91.0(84.0-99.0)	91.0(84.3-99.0)	88.0(82.0-97.0)	0.012*
Lp(a) (mg/dL)	4.7(2.3-11.7)	4.4(2.3-10.0)	15.4(3.9-29.3)	<0.001*
Hypertension (%)	11.1	11.1	10.9	ns
Diabetes mellitus (%)	13.7	14.1	9.2	ns
Smoking (%)	21.9	21.7	23.5	ns

**Figure 1:**
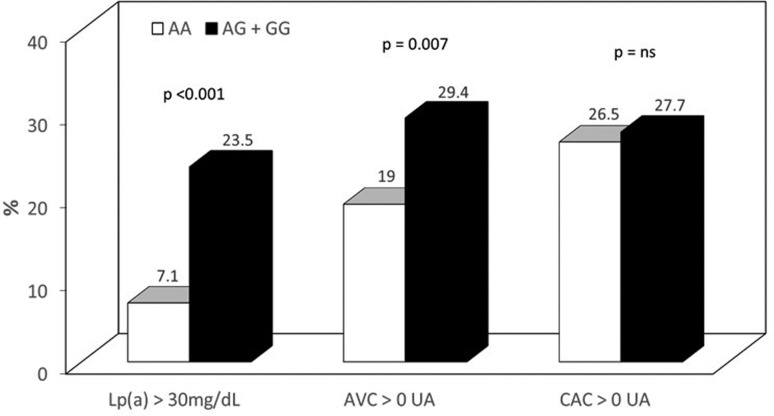
Prevalence of high Lp(a) levels and presence of aortic valve calcification (AVC) or coronary aortic calcification (CAC) with calcium score > 0 Hounsfield units (HU) in Mexican-Mestizo rs10455872-*G* allele carriers.

Subjects carrying the *G* allele (*AG*+*GG* genotypes) were of lower age (*p*=0.011). Anthropometric, physiological characteristics, as well as lipids, high and low-density lipoprotein concentrations were similar in both groups and not significantly different (Table 1). Compared with wild-type allele, *G* allele carriers showed a significantly higher Lp(a) plasma levels [15.4 (3.9-29.3 mg/dL vs. 4.4 (2.3-10.0 mg/dL), *p*<0.001, Table 1], higher prevalence of Lp(a) ≥ 30 mg/dL (23.5% vs. 7.1%, *p* < 0.001), and higher prevalence of AVC (29.4% vs. 19.0%, *p*=0.007, Figure 1). No significant differences were found in the prevalence of CAC > 0, or traditional risk factors, such as hypertension, diabetes mellitus, and smoking (Table 1, Figure 1).

**Figure 2:**
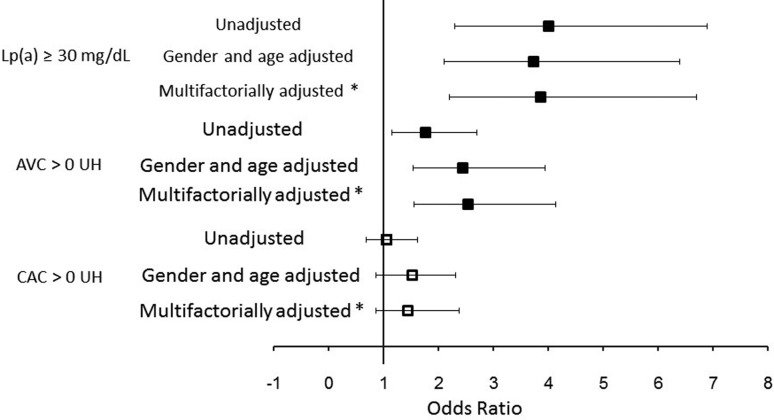
Multivariate regression analysis demonstrating the independent association between the *LPA* rs10455872-*G* allele and Lp(a) ≥ 30 mg/dL, AVC > 0 AU and CAC > 0 HU in a Mexican-Mestizo population. * adjusted for gender, age, BMI, triglycerides, LDL-C, diabetes mellitus, hypertension, and smoking.

The independence of the association between the rs10455872-*G* allele with the risk of presenting Lp(a) ≥ 30 mg/dL, CAC > 0, or AVC > 0 HU was investigated using multivariate logistic regression analysis models. Figure 2 shows the three statistical models used for each variable. The carriers of the *G* allele had a higher risk of presenting Lp(a) ≥ 30 mg/dL: OR = 4.01 (95% CI = 2.3 - 6.9, *p*<0.001), unadjusted model; OR = 3.73 (95% CI = 2.1 - 6.4, *p* < 0.001) in a model adjusted for gender and age; and OR = 3.86 (95% CI = 2.2 - 6.7, *p*=0.001) in a model adjusted for several coronary risk factors. The rs10455872-*G* allele was also associated with the risk of presenting AVC: unadjusted model, OR = 1.77 (95% CI = 1.16 - 2.70, *p*<0.008); gender and age adjusted model, OR = 2.45 (95% CI = 1.54 - 3.91, *p*<0.001); and OR = 2.54 (95% CI = 1.56 - 4.14, *p*=0.001) after adjusting for coronary risk factors. The rs10455872-*G* allele was not associated with CAC >0 HU (Figure 2).

## DISCUSSION

The most relevant result of this study was that, in Mexican-Mestizos without familiar and personal history of premature CAD, the *LPA* rs10455872-*G* allele was associated up to 3.86-times with values of Lp(a) ≥ 30 mg/dL and 2.54-times with the presence of AVC, independently of traditional CAD risk factors. Our data also demonstrate that subjects without clinical evidence of aortic valve disease, but carrying this variant of the *LPA* gene course with AVC, particularly those with high Lp(a) values (Bossé *et al.*, 2008; Thanassoulis *et al.*, 2013; Kamstrup *et al.*, 2014). It is interesting to note that two polymorphisms, rs6907156-*T* and rs7765803-*G*, that were associated with elevated Lp(a) plasma concentrations in Mexican-Mestizos, did not have effect on CAC or AVC. However, a future development of some inflammatory or chronic diseases (obesity, diabetes mellitus, or dyslipidemia) could impact on the association of Lp(a) with calcification process.

The concentration of Lp(a) in humans is genetically determined, and 20% to 90% of the variation in Lp(a) concentration can be explained by *LPA* gene polymorphisms (Dumitrescu *et al.*, 2011; Kamstrup *et al.*, 2014). The rs10444872-*G* allele of the *LP*A gene has been studied in a general population of different ethnic groups. In Denmark its prevalence is 14% (Glader *et al.*, 2003), 7% in European Caucasians (Stewart *et al.*, 1997; Thanassoulis *et al.*, 2013), it is below 1.0% in South Asia and in China (Lanktree *et al.*, 2010), and 2.0% in Afro-Americans and Latinos in the USA. However, for the same ethnic groups, the International Genome Sample Resource (IGSR) project, through 1000 GENOMES, reports frequencies below those described in the literature. These results point out the large differences in the prevalence of the rs10444872-*G* allele of the *LPA* gene among ethnic groups and the inconsistency of results obtained for a same group in different studies.

Information on the rs10444872-*G* allele of the *LPA* gene in the Mexican population was obtained from samples of Mexican-Americans that migrated to the USA from diverse regions of Mexico, mainly from rural zones were the proportion of an indigenous population is greater, which could explain the inconsistency of the results reported for Mexican-Americans that live in different areas of the USA (Haffner *et al.*, 1992; Kamboh *et al.*, 1997). The frequency of the *LPA* rs10455872-*G* allele found in the present study in Mexican-Mestizos was 9.4%, that is, 4.5-times higher than that informed in Latino or Afro-American populations in the USA, 9-times higher than in Asiatic groups; but lower than in Europeans or US Caucasians.

Some studies (Dumitrescu *et al.*, 2011; Kamstrup *et al.*, 2014; Santos *et al.*, 2014; Vongpromek *et al.*, 2015), have shown that patients with AVC present significantly elevated Lp(a) concentrations. However, in a recent comparison on the association of Lp(a) with AVC in four ethnic groups, this association was found significant in Caucasian and Afro-American subjects, but not in Hispanics or Chinese (Cao *et al.*, 2016). In contrast, the present results for the Mexican-Mestizo population reveal that there is a significant association of Lp(a) concentration with AVC, independent of other risk factors. The inconsistency of results could be explained by a selection bias, because the Mexican-Mestizo population studied was predominately urban.

The *LPA* gene polymorphisms have been associated with peripheral and coronary atherosclerosis (Mehrabi *et al.*, 2000), and recently, a large scale genetic meta-analysis showed the association of the rs10455872-*G* allele with AVC (Thanassoulis *et al.*, 2013). Similar results have been obtained in a general population, as well as in patients with CAD of different ethnic groups (Kamstrup *et al.*, 2014). All these results have been replicated in prospective studies performed in an open population (Dumitrescu *et al.*, 2011). Our results demonstrate that the rs10455872-*G* allele of the *LPA* gene is associated with the presence of AVC in a Mexican-Mestizo population. Few studies have approached simultaneously the association of *LPA* gene polymorphisms with Lp(a) concentrations and AVC in a general population. Recently, in a prospective study nested in the (EPIC)-Norfolk Study, patients with high Lp(a) and carriers of the rs10455872-*G* allele of gene *LPA* were found to have a higher risk of aortic valve stenosis, suggesting the possibility of a causal association (Arsenault *et al.*, 2014).

The mechanisms by which the aortic valve becomes mineralized are still unknown. It has been described that high concentrations of Lp(a) contain oxidized phospholipids, which, when hydrolyzed by phospholipase A2 associated with lipoproteins, generate lysophosphatidylcholine that has pro-inflammatory and osteogenic properties. Evidence in favor of this hypothesis is that both, oxidized phospholipids and lysophosphatidylcholine, have been found elevated in calcified aortic valves (Taleb *et al.*, 2011; Mahmut *et al.*, 2014). Another possible mechanism of aortic mineralization is related to the autotaxin enzyme, secreted by interstitial cells of the valve, which, by binding to Lp(a), uses as substrate the lysophosphatidylcholine present in the lipoprotein to generate phosphatidic acid, promoting thereby calcium hydroxyapatite deposition in the aortic valve (Bouchareb *et al.*, 2015). More recent research has revealed that lipid infiltration, inflammation, and osteogenesis are frequent pathogenic mechanisms involved in the valvular calcification process. In fact, oxidized LDL and metalloproteinases have been found associated with calcified stenotic valves (Edep *et al.*, 2000; Coté *et al.*, 2013). As a whole, these studies support the concept that aortic valve disease develops through a process similar to that of coronary atherosclerosis (Miller *et al.*, 2011; Bouchareb *et al.*, 2015).

The present study has the strength of including a large sample of a Mexican-Mestizo population without familial or personal antecedents of CAD or AVC that could bias the results of the analysis of the gene *LPA* polymorphisms, the prevalence of high Lp(a), or the presence of coronary or aortic valve calcification. To avoid inaccuracy in determining Lp(a) concentrations due to differences in apo(a) molecular mass, Lp(a) was determined through immunonephelometry with polyclonal antibodies that allow identifying both small and large isoforms of apo(a) (Marcovina *et al.*, 2000). It is important to emphasize that *G* allele carriers were of lower age, showing lower fasting glucose levels and lower prevalence of systemic hypertension and diabetes mellitus, which may support the probable causal relation between Lp(a) and valvular calcium deposition.

This study also has some limitations. First, by being cross-sectional, it does not allow establishing a causality relation, so it only allows making inferences. Second, as the sample was obtained from volunteers, participants may not represent the general population. However, it would be expected that the risk relation would be similar to that of a randomized sample, because of the improbability that the participants in the study could have a previous knowledge on the calcification of their coronary artery, aortic valve, or *LPA* genotype. In addition, the prevalence of traditional risk factors observed in our study is similar to that reported in the ENSANUT survey of national representation (Hernández-Avila M *et al.*, 1998). Third, in this study, we did not perform hemodynamic measurements, and hence, we are unable to estimate the possible presence of aortic stenosis, although this alteration is quite improbable considering the low Hounsfield scores found in the studied population.

## Conclusions

Our study performed on an asymptomatic Mexican-Mestizos population demonstrates a significant and independent association of the rs10455872-*G* allele of the *LPA* gene with high Lp(a) levels and with the presence of AVC. In this ethnic group, the Lp(a) prevalence higher than 30 mg/dL, coronary artery calcification, and the presence of aortic valvular calcium were of 9.2%, 26.6%, and 20%, respectively. Our data point out the need to perform longitudinal studies that would allow characterizing the role of *LPA* gene polymorphisms as genetic markers for high Lp(a) levels and elevated risk for aortic valvular calcification in Mexicans.
